# Three factor delay learning rules for spiking neural networks

**DOI:** 10.3389/fnins.2026.1814505

**Published:** 2026-05-20

**Authors:** Luke Vassallo, Nima Taherinejad

**Affiliations:** ECLECTX Team, Institute of Computer Engineering, Heidelberg University, Heidelberg, Germany

**Keywords:** neuromorphic computing, online learning, spiking neural networks (SNNs), synaptic and axonal delays, three-factor learning rules

## Abstract

Spiking neural networks (SNNs) are hybrid dynamical systems that operate on spatiotemporal data, yet their learnable parameters are often limited to synaptic weights, contributing little to temporal pattern recognition. Learnable parameters that delay spike times can improve classification performance in temporal tasks, but existing methods rely on large networks and offline learning, making them unsuitable for real-time operation in resource-constrained environments. In this paper, we introduce synaptic and axonal delays to leaky integrate and fire (LIF)-based feedforward and recurrent SNNs, and propose three-factor learning rules to simultaneously learn weights and delays online. We employ a smooth Gaussian surrogate to approximate spike derivatives exclusively for the eligibility trace calculation, and together with a top-down error signal determine parameter updates. Our experiments show that incorporating delays improves accuracy by up to 18% over a weights-only baseline, and for networks with similar parameter counts, jointly learning weights and delays yields up to 14% higher accuracy. On the SHD speech recognition dataset, our method achieves similar accuracy to offline backpropagation-based approaches. Compared to state-of-the-art methods, it reduces model size by 6.6× and inference latency by 50%, with only a 2.5% drop in classification accuracy. Our findings would be beneficial for the design of power and area-constrained neuromorphic processors by enabling on-device learning and lowering memory requirements.

## 1 Introduction

SNNs are hybrid dynamical systems whose internal state is described by continuous time dynamics, namely integrating the weighted sum of action potentials, and a discontinuous threshold mechanism that emits discrete spikes. Unlike artificial neural networks (ANNs), which perform static data transformations without an explicit temporal component, SNNs inherently operate in the time domain. However, despite this fundamental difference, SNNs still rely primarily on synaptic weights for learning (Bittar and Garner, 2022; Yin et al., 2021). The absence of dedicated mechanisms for capturing temporal dynamics often leads to lower task performance, as shown by state-of-the-art methods for learning temporal delays (Mészáros et al., 2025; Hammouamri et al., 2024; Sun et al., 2023). Alternatively, achieving competitive performance often requires significantly larger models with orders of magnitude more parameters (Bittar and Garner, 2022). Recently improved techniques for training SNNs such as surrogate gradient methods (Neftci et al., 2019), have allowed learning of time-dependent parameters on a large scale such as synaptic delay learning.

**Figure 1:**
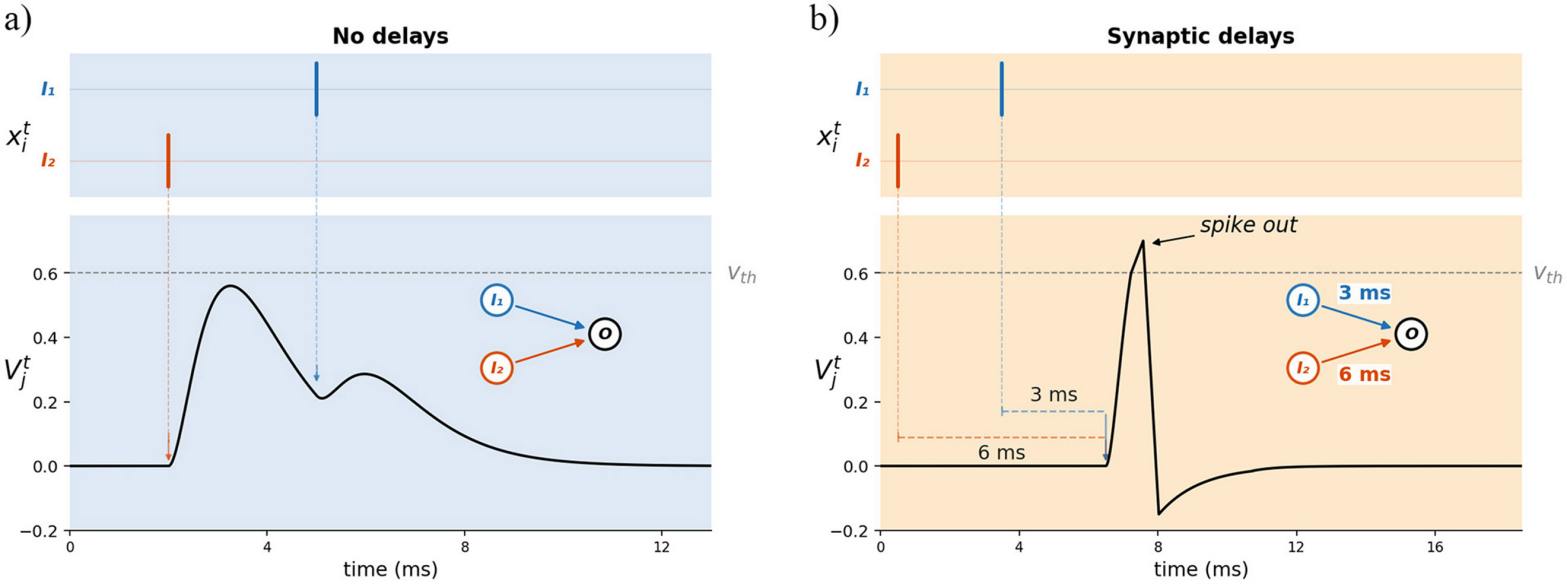
Effect of synaptic delays on post-synaptic integration. Two pre-synaptic neurons *I*_1_ and *I*_2_ project to output neuron *O* with an identical 3 ms firing offset in both panels. **(a)** Without delays, postsynaptic potentials arrive apart, fail to summate above threshold, and no spike is produced. **(b)** Synaptic delays compensate the firing offset, causing coincident postsynaptic potentials arrival, and an output spike. Dashed arrows indicate each spike's propagation through its synaptic delay to the moment of membrane influence.

Learnable synaptic delays add a degree of freedom that facilitates temporal pattern recognition. As shown in Figure 1, a delay applied to a synaptic connection shifts the temporal influence of a pre-synaptic spike at the post-synaptic membrane, effectively acting as a learnable phase shift in the input space.When delays are tuned such that inputs from different pre-synaptic neurons arrive coincidentally, downstream neurons can fire— a computation that is impossible through synaptic weights alone when the underlying spike timing is unfavorable. The same mechanism also works in reverse: delays can spread otherwise coincident inputs apart, suppressing unwanted firing. Together, this gives a network fine-grained control over which temporal patterns of input activity drive output spikes and which do not.

Offline learning methods achieve high accuracy, but as their memory requirements scale with the sequence length, their suitability for edge deployment becomes challenging. Recent works (Bèna et al., 2025; Göltz et al., 2025; Pehle et al., 2023) show promise in alleviating these constraints under sparse spiking conditions, but it is still an open question whether they can meet the demands of edge applications operating with limited energy, memory and compute resources. By releasing the memory constraints, online learning methods emerge as an enabler for integrating learning on-device and can thus improve task accuracy in applications with non-stationary data or those operating in dynamic environments. Numerous applications benefit from these features, including wearable systems, brain–computer interfaces, and speech recognition applications.

Our work introduces three-factor delay learning rules for updating synaptic and axonal delays using eligibility propagation (Bellec et al., 2020) as the underlying learning algorithm. We explore the application of these rules to keyword spotting (KWS) tasks using feedforward SNNs and spiking recurrent neural networks (SRNNs). Our results show that synaptic delays can be learned with effectiveness close to that achieved with backpropagation (BP), while axonal delays can also be learned, though less effectively. Consistent with prior literature, learnable delays provide little benefit in dense networks but improve performance in smaller or sparse networks. Our proposed method, along with its systematic evaluation, provides insights for chip designers balancing model versatility and efficiency in size weight and power (SWaP)-constrained neuromorphic processors.

## 2 Background

**Figure 2:**
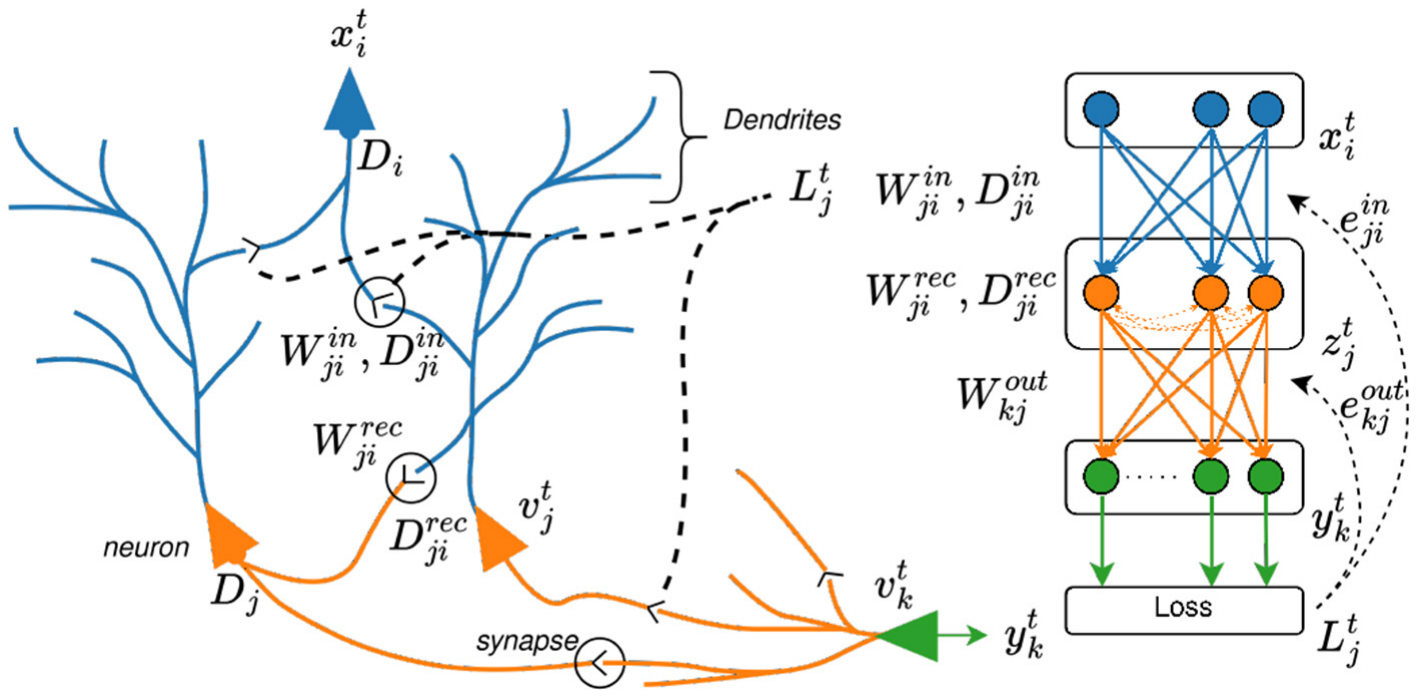
Morphological view of a subset of the neurons with synaptic connections along the dendritic arbor, traces showing the effect of delays, and spike representation for gradient based learning. Adjacent is an equivalent simplified logical view of the SNN.

Delays in point neurons can be categorized based on morphology (see Figure 2). At the synapse, delays arise from the complex chemical reactions triggered by the arrival of an action potential at the pre-synaptic site, which later initiates the post-synaptic response (Sabatini and Regehr, 1996). If the post-synaptic response causes the neuron to fire, the resulting spike propagates along the axon introducing an axonal delay (Debanne et al., 2011). Before reaching the synapse of a downstream neuron, the signal must also traverse the dendritic arbor, leading to a dendritic delay that depends on dendritic morphology, membrane properties, and the presence of active ion channels (London and Häusser, 2005).

### 2.1 Delay learning

Several approaches exist to embed and learn delays in SNNs, which can be broadly grouped into methods that compute exact gradients and those that use surrogate gradients to approximate derivatives across discontinuous components.

Exact methods compute analytical gradients of the loss with respect to weights and delays directly from spike timings. DelGrad (Göltz et al., 2025) derives closed form gradients from the spike response model. Similarly, methods based on event propagation Wunderlich and Pehle (2021) formulate SNNs in continuous time and compute gradients using the adjoint method, where adjoint variables evolve between spikes and jump conditions, propagate gradients across events. Event-based delay learning has recently been extended to heterogeneous and recurrent delays Mészáros et al. (2025), and together with appropriate loss shaping achieves near state-of-the-art performance on keyword-spotting benchmarks Nowotny et al. (2025) with high temporal resolution.

Surrogate-gradient-based methods (Neftci et al., 2019) provide a practical approach to optimizing SNNs in the presence of non-differentiable spike dynamics, and have been widely applied to learning synaptic and axonal delays. Approaches such as spike layer error reassignment in time (SLAYER) (Shrestha and Orchard, 2018) address temporal credit assignment by propagating errors backward through spike times using response kernels, enabling joint learning of synaptic weights and delays. Concurrently, Deckers et al. (2024) show that combining delays with adaptive leaky integrate and fire (ADLIF) enables smaller networks to achieve competitive performance compared to standard ADLIF Bittar and Garner (2022). However, recent work on SE-adLIF (Baronig et al., 2025) demonstrates near state-of-the-art accuracy without explicit delays, raising the question on the conditions under which delay learning can provide complementary benefits to learnable adaptive thresholds. Kernel-based formulations further extend this paradigm by parameterizing delays as continuous temporal convolutions over spike trains, for learning axonal delays using neurophysiologically inspired models (Sun et al., 2023, 2022) or a more general interpolation-based method of dilated convolution with learnable spacings (DCLS) (Khalfaoui-Hassani et al., 2023) that enable gradient-based optimization of delay parameters, the latter providing a more flexible approach leveraging the PyTorch autograd engine and applicable to both feedforward (Hammouamri et al., 2024; Mészáros et al., 2024) and recurrent networks (Queant et al., 2025).

### 2.2 Online learning

Online learning in SNNs involves processing input sequences while simultaneously collecting spatially and temporally local information needed to compute parameter updates. These methods aim to avoid both the high memory requirements of backpropagation through time (BPTT) and its lack of biological plausibility. In particular, BP suffers from update locking (Jaderberg et al., 2017), preventing any parameter updates until an entire input sequence has been processed. For sequential data, this leads to memory usage that grows linearly with sequence length, limiting the feasibility of BPTT in real-time or resource-constrained settings.

While some online learning approaches are grounded in Hebbian principles, our focus is on three-factor learning rules. These rules address key sources of biological implausibility in BPTT-like algorithms (Rumelhart et al., 1986; Wunderlich and Pehle, 2021), including the weight-transport (Liao et al., 2016) and update-locking (Jaderberg et al., 2017) problems. They rely on eligibility traces that encode the temporally filtered co-activity of pre- and post-synaptic units. Unlike two-factor learning rules (Bi and Poo, 1998), synaptic updates occur only when these traces are gated by a third, modulatory signal—typically a top-down learning signal that conveys task-relevant error, reward, or surprise information (see Marschall et al. (2020) for a comprehensive survey). Three-factor approaches trace their origins to real-time recurrent learning (RTRL) (Williams and Zipser, 1989), which computes gradients of hidden states online and thus avoids unrolling the network over a time window *T*. However, its cubic complexity ($O\left(n^{3}\right)$) makes it substantially more costly than BPTT (O(nT)), motivating subsequent work toward scalable approximations (Benzing et al., 2019; Tallec and Ollivier, 2018) and biologically plausible variants (Gerstner et al., 2018).

Superspike (Zenke and Ganguli, 2018) constructs eligibility traces by combining a low-pass filtered presynaptic trace with a non-linear surrogate of post-synaptic activity, while using an error signal as the modulatory factor. Eligibility propagation (e-prop) (Bellec et al., 2020) reformulates BPTT within a three-factor framework, closely approximating its performance in both feedforward and recurrent architectures. Dynamics for deep continuous local learning (DECOLLE) (Kaiser et al., 2020) generates local synthetic gradients using layer-wise readouts, enabling fully online updates. Finally, the method introduced in (Bohnstingl et al., 2022) separates spatial gradients (top-down signals within each timestep) from temporal gradients (eligibility traces across timesteps). This formulation is equivalent to BPTT for shallow SRNNs and achieves competitive approximations for deeper networks.

Existing research demonstrates benefits from treating delays as learnable parameters, particularly in structurally sparse spiking networks. Power-constrained applications requiring adaptation (e.g., speech recognition and biomedical signal processing) can benefit from adjusting these parameters while maintaining a tractable memory and computational footprint. The algorithm proposed in the next section uses eligibility propagation to derive three-factor learning rules for updating delay parameters.

## 3 Materials and methods

We present the neural dynamics for point neurons, network architecture, and three-factor delay learning rules using e-prop (Bellec et al., 2020) as a basis for online learning.

### 3.1 Neuron dynamics

The LIF model stands out for its computational efficiency and biological plausibility, and has become the defacto standard model in neuromorphic engineering (Abbott, 1999; Gerstner et al., 2014). For a point neuron *j* the LIF model integrates the weighted sum of input spikes into a one-dimensional hidden variable $v_{j}^{t}$, representing the membrane potential. When the membrane potential exceeds a pre-defined threshold, *v*_*th*_ the neuron generates an action potential or spike, $z_{j}^{t}\in \left\{0,1\right\}$, which propagates along its axon. The model consists of two components namely, a first-order linear differential equation describing the evolution of the membrane potential and a spike generation mechanism. The leaky integration of synaptic currents into the membrane potential *v*_*j*_(*t*) is given by


$$\begin{array}{l} \tau_{m}\frac{dv_{j}}{dt}=-\left(v_{j}\left(t\right)-v_{reset}\right)+RI_{j}\left(t\right) \end{array}$$ (1)

where τ_*m*_ is the membrane time constant, *v*_*reset*_ is the membrane potential at rest, *R* is the input resistance, and *I*_*j*_(*t*) is the input synaptic current at time, *t*. For simplicity, the synaptic current described by Equation 2 is assumed stateless, and represented as a weighted sum between the synaptic weight, $W_{ji}^{in}$ and the delayed pre-synaptic input $x_{i}\left(t-D_{ji}^{in}\right)$. The synaptic $D_{ji}^{in}\in \mathbb{Z}$, or $D_{i}^{in}\in \mathbb{Z}$ axonal delay is described in number of timesteps, bounded by *D*_*max*_.


$$\begin{array}{l} I_{j}\left(t\right)=\underset{i}{\sum }W_{ji}^{in}x_{i}\left(t-D_{ji}^{in}\right) \end{array}$$ (2)

Equation 1 is solved analytically under the assumptions *v*_reset_ = 0 and $R={\left(1-e^{-\frac{\Delta t}{\tau_{m}}}\right)}^{-1}$. The solution is discretized with a timestep Δ*t*, yielding the discrete timestepped neuron dynamics in Equation 3:


$$\begin{array}{l} v_{j}^{t+1}=\alpha v_{j}^{t}+\underset{i}{\sum }W_{ji}^{\text{in}}x_{i}^{t-D_{ji}^{in}} \end{array}$$ (3)

where $\alpha =e^{-\frac{\Delta t}{\tau_{m}}}$ is the decay factor determined by the membrane time constant τ_*m*_ and the timestep Δ*t*.

The spike reset mechanism extends the membrane potential dynamics with a thresholding operation, mathematically represented using the Heaviside function:


$$\begin{array}{l} z_{j}^{t+1}=H\left(v_{j}^{t}>v_{th}\right) \end{array}$$ (4)

where $z_{j}^{t+1}$ is a binary variable indicating the presence of a spike at time *t*+1. Following the spike event, the membrane potential is explicitly reset by deducting *v*_*th*_. The full dynamics for an SNN are thus given by:


$$\begin{array}{l} v_{j}^{t+1}=\alpha v_{j}^{t}+\underset{i}{\sum }W_{ji}^{in}x_{i}^{t-D_{ji}^{in}}-z_{j}^{t}v_{th} \end{array}$$ (5)

The neuron dynamics can be extended with recurrent connections $W_{ji}^{rec}$ and optional axonal $D_{j}^{rec}$ or synaptic $D_{ji}^{rec}$ delays resulting the SRNN formulation shown in Equation 6.


$$\begin{array}{l} v_{j}^{t+1}=\alpha v_{j}^{t}+\underset{i}{\sum }W_{ji}^{rec}z_{i}^{t-D_{ji}^{rec}-1}+\underset{i}{\sum }W_{ji}^{in}x_{i}^{t-D_{ji}^{in}}-z_{j}^{t}v_{th} \end{array}$$ (6)

### 3.2 Neural network architecture and loss function

The neural network architecture, shown in Figure 2, comprises a set of virtual input neurons, followed by a single hidden layer of LIF neurons with optional recurrent connections, and a final readout layer. The virtual input neurons deliver the spiking input signal, optionally incorporating axonal delays. The hidden layer is parameterized by synaptic weights and, when enabled, synaptic delays. Its neural dynamics follow Equations 5, 6 for SNNs and SRNNs respectively. The output of the LIF hidden layer projects to a readout layer composed of leaky integrate (LI) neurons, whose dynamics are described by Equation 3 but without the spike-reset mechanism. The leakage of the readout neurons is defined by $\kappa =e^{-\frac{\Delta t}{\tau_{o}}}$, where τ_*o*_ is the membrane time constant. In practice, a large membrane time constant ( τ_*out*_≥1*s*) is assigned to the LI neurons in the readout layer, enabling them to effectively maintain their state over time. Note that no delays are applied in the readout layer, as their influence on the final classification would be negligible.

For k-class classification, we assume *k* categories represented as a K-dimensional one-hot encoded vector. A softmax function is applied to the output from the readout layer, and the cross entropy loss is then computed $E=-\underset{k}{\sum }\pi_{k}\underset{t}{\sum }log\left({\hat{\pi }}_{k}^{t}\right)$, where π_*k*_ represents the ground-truth classification label for class *k* and ${\hat{\pi }}_{k}^{t}$ denotes the corresponding probability predicted by the network at time *t*.

#### 3.2.1 Surrogate gradient

The spiking mechanism described by Equation 4 is discontinuous and therefore non-differentiable. It blocks the flow of gradient information and is effectively replaced by means of a surrogate function used only during the calculation of parameter updates. We adopt the piecewise linear function in Equation 7 with γ_*pd*_ = 0.3.


$$\begin{array}{l} \frac{dz}{dv_{j}^{t}}\approx \frac{\gamma_{pd}}{v_{th}}\max\left(0,1-\left|\frac{v_{j}-v_{th}}{v_{th}}\right|\right) \end{array}$$ (7)

### 3.3 Online learnable delays

Eligibility propagation (e-prop) (Bellec et al., 2020) is an online learning algorithm that closely approximates BPTT while describing synaptic weight updates as three-factor learning rules. The formulation supports delay-parametrized spike trains but does not permit the delays to be learnable. This work proposes the three-factor learning rules in Equation 8 using the same refactorization to calculate error gradients with respect to network parameters describing temporal delays. $\frac{dE}{dD_{ji}}$ represents the error gradients with respect to synaptic delays $D_{ji}\in \left\{D_{ji}^{in},D_{ji}^{rec}\right\}$. Similarly, $\frac{dE}{dD_{i}}$ corresponds to the gradient with respect to axonal delays $D_{i}\in \left\{D_{i}^{in},D_{j}^{rec}\right\}$.


$$\begin{array}{ll} \frac{dE}{dD_{ji}} & =\underset{t}{\sum }\frac{dE}{dz_{j}^{t}}\cdot {\left[\frac{dz_{j}^{t}}{dD_{ji}}\right]}_{\text{local}} \end{array}$$ (8)

The top down learning signal $L_{j}^{t}=\frac{dE}{dz_{j}^{t}}$ is derived from the cross-entropy loss function applied to the readout layer outputs defined in Section 3.2. The resulting expression is given in Equation 9:


$$\begin{array}{l} \frac{dE}{dz_{j}^{t}}=\underset{k}{\sum }W_{kj}^{out}\underset{t^{\prime }\geq t}{\sum }\left(\pi_{k}^{t^{\prime }}-\pi_{k}^{*,t^{\prime }}\right)\kappa^{t^{\prime }-t} \end{array}$$ (9)

The eligibility trace $e_{ji}^{t}=\frac{\partial z_{j}^{t}}{\partial v_{j}^{t^{\prime }}}$ incorporates information about the previous spiking activity and can be recursively expressed, permitting real-time implementation as shown in Equation 10. Here, $\frac{\partial z_{j}^{t}}{\partial v_{j}^{t^{\prime }}}$, is the surrogate gradient of the neuron's output with respect to the hidden state calculated with Equation 7, $\frac{\partial v_{j}^{t}}{\partial v_{j}^{t-1}}=\alpha$ is computed by differentiating Equation 5 with respect to $v_{j}^{t}$, and $\epsilon_{ji}^{t-1}$ is the eligibility vector.


$$\begin{array}{l} e_{ji}^{t}=\frac{\partial z_{j}^{t}}{\partial v_{j}^{t^{\prime }}}\left(\frac{\partial v_{j}^{t}}{\partial v_{j}^{t-1}}\cdot \epsilon_{ji}^{t-1}+\frac{\partial v_{j}^{t}}{\partial D_{ji}}\right) \end{array}$$ (10)

Calculating the eligibility trace entails computing the derivative of the hidden state described by Equation 6 with respect to the delay parameters, $\frac{dv_{j}^{t}}{dD_{ji}}$. However since *D*_*ji*_ is associated with spike trains (input $x_{i}^{t-D_{ji}^{in}}$ or recurrent $z_{i}^{t-D_{ji}^{rec}}$) the derivative does not exist. We overcome this problem and approximate the derivative by using surrogate gradients.

### 3.4 Delay surrogate gradient

By assuming that a spike has a continuous profile, we can compute its derivative analytically and thereby approximate the gradient $\frac{dv_{j}^{t}}{dD_{ji}}$. This approach is well-established in the literature, for example, Wang et al. (2019) employs a Laplacian kernel to model the spike shape, while Hammouamri et al. (2024) uses a Gaussian kernel, both non-causal and symmetrical around the spike.

A Gaussian kernel was selected based on preliminary experiments comparing causal exponential kernels with anti-causal double-exponential and symmetric Gaussian kernels. From an implementation standpoint, causal kernels can lower padding requirements by half, however, when used as surrogates during training, they failed to achieve convergence. Although gradient information propagates through the network, updates tend to be biased in one temporal direction, potentially destabilizing learning through exploding gradients. In contrast, anti-causal or symmetric kernels distribute gradient information more uniformly around the spike threshold, enabling more stable optimization. Notably, neither the precise temporal width of the kernel nor its smoothness had a major effect on performance, while symmetry emerged as the more critical factor for learning delays. Similar effects have been discussed in the literature (Neftci et al., 2019; Zenke and Vogels, 2021) in the context of neuron thresholds, though here they apply directly to the spike itself.

Equation 11 describes a spike train parametrized with synaptic delay *D*_*ji*_ as a sum of *k* dirac delta functions delayed in time by *t*_*k*_+*D*_*ji*_,


$$\begin{array}{l} x_{i}^{t-D_{ji}}=\underset{k}{\sum }\delta \left(t-t_{k}-D_{ji}\right) \end{array}$$ (11)

where δ is the Dirac delta function, *t*_*k*_ are the spike times, and *D*_*ji*_ is the synaptic delay between the pre-synaptic neuron *j* and the post-synaptic neuron *i*. The Dirac delta function is approximated with a Gaussian and parameterized by the synaptic delay, *D*_*ji*_ (*D*_*i*_ for axonal delay), where $D_{ji}=-\frac{D_{max}-1}{2}$ represents no delay and $D_{ji}=\frac{D_{max}-1}{2}$ represents the maximum delay, similar to Hammouamri et al. (2024). Equation 12 describes the delay parametrized Gaussian kernel. During parameter updates, *D*_*ji*_ is clamped within $\pm \frac{D_{max}-1}{2}$.


$$\begin{array}{l} x_{i}^{t-D_{ji}}\approx \frac{1}{\sqrt{2\pi }\sigma }e^{-\frac{{\left(t-t_{k}-D_{ji}\right)}^{2}}{2\sigma^{2}}} \end{array}$$ (12)

The derivative of Equation 12 with respect to the delay parameters can now be calculated, yielding Equation 13:


$$\begin{array}{l} \frac{dx_{i}^{t-D_{ji}}}{dt}=-\frac{t-t_{k}-D_{ji}}{\sqrt{2\pi }\sigma^{3}}e^{-\frac{{\left(t-t_{k}-D_{ji}\right)}^{2}}{2\sigma^{2}}} \end{array}$$ (13)

**Figure 3:**
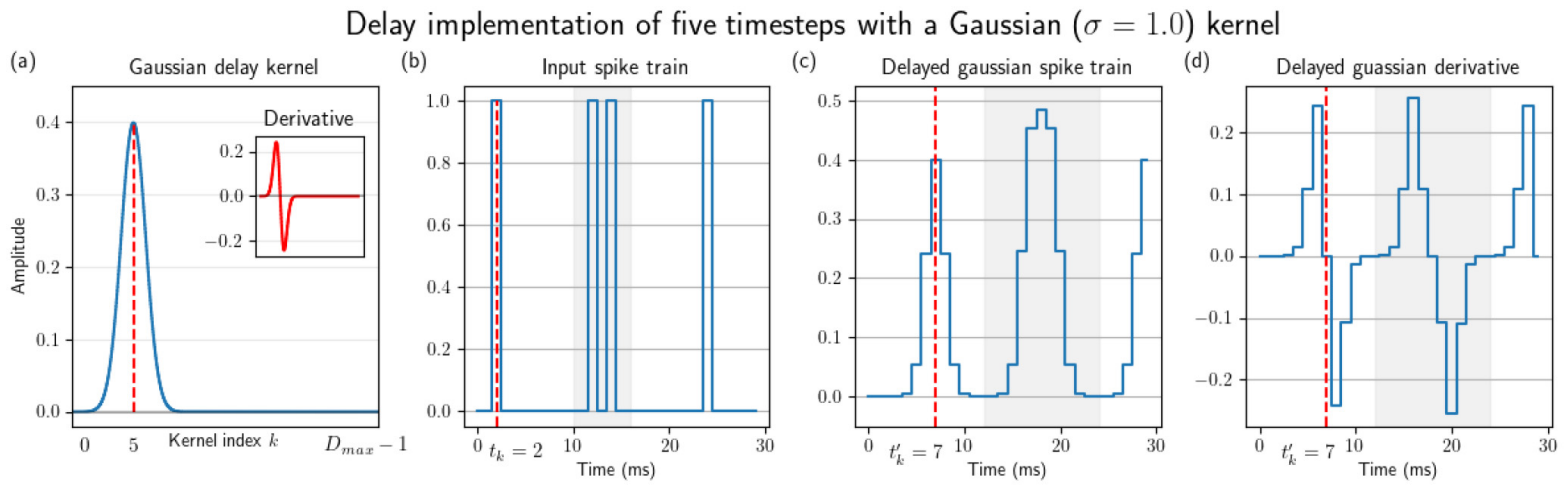
Gaussian kernel and its application in delaying spike trains and computing their derivatives. **(a)** Gaussian kernel with a delay of five timesteps and its derivative (shown in the inset), **(b)** an arbitrary spike train **(c)** the delayed spike train fitted with a Gaussian function-spike at *t*_*k*_ = 2 is now delayed by five timesteps to $t_{k}^{\prime }=7$, and **(d)** its corresponding delayed derivative.

Figure 3 illustrates the application of the Gaussian kernel for a delay of five timesteps. The Gaussian kernel's symmetry around the spike response may be truncated when applied without padding. This issue is particularly relevant at the extremities of the temporal boundaries—specifically for very small delays or for delays approaching the maximum allowable value. For instance, in Figure 3d, a large delay results in the portion of the kernel preceding the spike being truncated.

The adoption of a Gaussian kernel does not impact the ability to calculate gradients online, and only introduces a practical limitation. Because the kernel is non-causal, the surrogate spike derivative depends on membrane potentials occurring both before and after the spike time. This dependence will require additional buffering for its implementation, equal to half of the size of the kernel. However, since the size of the kernel is often much smaller than the maximum delay and scales per neuron, the impact should be low overall (see Section 6).

## 4 Experiments

Using KWS tasks (Cramer et al., 2022), we systematically evaluate the proposed three-factor delay learning rules against offline methods (Hammouamri et al., 2024) using dense and structurally-sparse SNNs. The experiments extend to SRNNs, with ablation tests to identify where delays impact task performance. Hammouamri et al. (2024) DCLS-based method was selected as a baseline because it uses an LIF model and achieves state-of-the-art performance using temporal convolutions together with BPTT. Synaptic delays are introduced at input synapses for SNNs and additionally at recurrent synapses for SRNNs. Axonal delays are applied to virtual input neurons for SNNs and to both input and recurrent neurons for SRNNs.

### 4.1 Dataset

We use the spiking heidelberg digits (SHD) and spiking speech commands (SSC) datasets to evaluate our implementation of online delay learning. Both datasets consist of spoken sequences in spiking format, generated by preprocessing the original audio using an artificial cochlear model, which converts the auditory signals into spikes (Cramer et al., 2022).

The SHD dataset consists of 10,000 professionally recorded samples from 12 participants and contains spoken sequences of the digits zero through nine in both English and German. SSC dataset is derived from the speech commands (SC) dataset (Warden, 2018) and contains 100,000 samples of 35 commonly spoken keywords collected from a diverse group of participants under varying recording conditions.

The datasets consist of 700 spiking neurons with a temporal resolution of 1 ms. To reduce computational time and GPU memory requirements, the spatial dimension is binned by a factor of six, resulting in 700/6 = 116 input neurons. Additionally, the temporal dimension is sub-sampled at a rate of 10 ms yielding sequences of approximately 100 samples in length.

### 4.2 Experimental setup

We evaluate online delay learning in SNNs and SRNNs across three configurations: a fully connected network with 128 hidden neurons, an 80% sparse variant, and a small fully connected network with 16 hidden units. Sparsity is implemented with fixed random binary maps.

SNN experiments are complemented by evaluating the same configurations using BPTT-based DCLS, and for this, we extend the state-of-the-art Hammouamri et al. (2024) with axonal delays. The online learning experiments are repeated using SRNNs with both input and recurrent delays (DCLS experiments are omitted because offline implementation is unsupported for SRNNs). Evaluations on the SSC dataset follow a similar but less exhaustive strategy, for several reasons. The computational demands are considerably greater, training times are substantially longer owing to the dataset's size, and memory constraints necessitate small batch sizes, which further compounds this cost. Hidden layer width was therefore limited to 256 fully connected neurons, though both feedforward and recurrent variants are still explored. Furthermore, state-of-the-art performance on this dataset typically favors deep architectures, which lies outside the scope of what our model support that tends to favor smaller tasks that can be accommodated with a single versatile hidden layer.

Learnable delays tend to offer a smaller contribution compared to weights Hammouamri et al. (2024); Mészáros et al. (2024). To assess the efficacy of delay learning, we perform control experiments. For SNNs, we evaluate delay learning as a function of sparsity under fixed and co-learned conditions. We perform similar experiments for SRNNs but additionally analyze the effect of placing delays at the input, in the recurrent connections, or both.

The learning rates for weights and delays are set to 10^−4^ and 10^−2^, respectively. Training is conducted with a batch size of 16 samples over 60 epochs for both the SHD dataset and SSC datasets. Synaptic and axonal delays are limited to 25 timesteps thereby introducing a maximum latency of 250ms. The inference pass uses binary spike trains and the Gaussian kernel is only invoked during parameter updates. Offline DCLS-based experiments retain all original hyperparameters (Hammouamri et al., 2024). We train all networks on the training dataset and evaluate top-1 classification accuracy on the test set. Each experiment is repeated five times, and test accuracy is reported with a 95% confidence interval, assuming a t-distribution. The implementation is performed in PyTorch 2.1 and runs on a Debian 12 system with a Xeon 6526Y CPU, 1 TB of memory, and a 20 GB RTX A4500 Ampere GPU.

## 5 Results

Table 1 reports the top-1 test accuracy on the SHD dataset for the SNN and SRNN configurations described in Section 4.2. Comparing the DCLS-based baseline with our proposed method for fully connected weights-only models in rows one and four respectively, reveals a 15% improvement in favor of our method. The underlying neuron model is identical in both setups and thus the observed difference is likely due to variations in implementation and hyperparameters. One key distinction is that our method does not use PyTorch's autograd engine and, after computing the gradients as described in Section 3.4, it manually updates the gradient fields at every timestep. Additional differences arise from the choice of hyperparameters, in particular the decay rate for our implementation is set to $e^{-\frac{dt}{\tau_{m}}}=0.6$, while for the baseline SpikingJelly implementation (Fang et al., 2023) uses $\alpha =1-\frac{1}{\tau_{mem}}=0.5$ $=1-\frac{1}{\tau_{mem}}=0.9$. For configurations with synaptic and axonal delays, our implementation achieves test accuracies that closely match the BPTT-based baseline (within 0.15%) for fully connected models. Both algorithms show that, compared to a weights-only model, the presence of axonal delays, a mere addition of 128 parameters, contributes the majority of the increase in accuracy, and the addition of synaptic delays contributes no further than 1% to classification accuracy while almost doubling the number of parameters. In our model, synaptic and axonal delays yield increases of 13.2% and 12.6% in test classification accuracy, respectively, compared to a weights-only baseline. DCLS-based models have a larger gap due to the lower accuracy of the weights-only model, but nevertheless both methods reach the same top-1 classification accuracy. These results highlight the importance of temporal parameters in SNNs for evoking complex post-synaptic responses across time.

**Table 1:** Test classification accuracy for SNN and SRNN models trained with online and offline (BP) methods.

ID	Dataset	Configruation	Weights only		Weights, fixed synaptic delays (LW+F-SD)	Weights, learnable synaptic delays (LW+L-SD)		Weights, fixed axonal delays (LW+F-AD)	Weights, learnable axonal delays (LW+L-AD)	
			Accuracy (%)	Params (k)	Accuracy (%)	Accuracy (%)	Params (k)	Accuracy (%)	Accuracy (%)	Params (k)
1	SHD	FC^†^-128 SNN (BP^§^)	63.70% ± 1.6%	17.4	90.19% ± 0.27%	92.79% ± 1.1%	32.3	89.32% ± 0.6%	91.81% ± 2.14%	17.5
2		SP^‡^-128 SNN (BP)	59.72% ± 3.9%	3.5	84.27% ± 3.32%	88.42% ± 2.7%	6.5	81.73% ± 3.61%	85.82% ± 3.27%	3.5
3		FC-16 SNN (BP)	50.33% ± 6.8%	2.2	76.07% ± 3.79%	82.79% ± 1.0%	4.0	74.36% ± 8.81%	79.6% ± 17.96%	2.3
4		FC-128 SNN	79.46% ± 0.2%	17.4	92.42% ± 0.22%	92.64% ± 0.4%	32.3	91.90% ± 0.4%	92.01% ± 0.3%	17.5
5		SP-128 SNN	71.23% ± 1.4%	3.5	86.26% ± 0.79%	89.22% ± 0.8%	6.5	86.36% ± 0.6%	88.06% ± 2.2%	3.5
6		FC-16 SNN	69.09% ± 1.4%	2.2	83.87% ± 2.47%	85.58% ± 4.3%	4.0	84.93% ± 1.4%	84.92% ± 0.7%	2.3
7		FC-128 SRNN	85.77% ± 0.94%	33.8	91.73% ± 0.81%	92.36% ± 0.3%	65.0	91.66% ± 0.6%	91.64% ± 1.6%	34.0
8		SP-128 SRNN	83.52% ± 2.38%	6.76	90.18% ± 0.20%	91.40% ± 0.71%	13.00	89.27% ± 1.40%	90.42% ± 0.39%	6.81
9		FC-16 SRNN	73.29% ± 1.02%	2.43	83.92% ± 5.80%	85.81% ± 2.88%	4.54	84.09% ± 4.32%	83.49% ± 6.58%	2.56
10	SSC	FC-256 SNN (BP)	43.16% ± 0.16%	34.82	69.67% ± 0.10%	72.87% ± 0.03%	64.51	69.18% ± 0.12%	71.54% ± 0.15%	34.93
11		FC-256 SNN	52.24% ± 0.05%	34.82	70.93% ± 0.24%	71.90% ± 0.06%	64.51	69.35% ± 0.48%	70.18% ± 0.04%	34.93
12		FC-256 SRNN	68.86% ± 0.05%	100.35	73.52% ± 0.01%	74.66% ± 0.09%	195.58	72.37% ± 0.14%	73.18% ± 0.07%	100.72

^†^ FC, Fully Connected; ^‡^ SP, Sparse (80%); ^§^ BP, Backpropagation.

One hundred and twenty-eight-wide fully connected models do not show significant improvement from delay learning. This is shown by the negligible increase in test-classification accuracy between configurations that learn weights and those that jointly learn weights-and-delays. In contrast, smaller models—obtained either through fixed-binary sparsity masks zeroing 80% of the connections (rows two and five), or by using a narrow albeit fully connected hidden layer (rows three and six)—demonstrate that, under these conditions, learning delays can make a meaningful contribution. In fact, synaptic delays contribute 3% and 1.7% while axonal delays contribute 2.3% and no improvement for sparse and small fully-connected models respectively. The ability to learn delays online offers practical advantages for neuromorphic hardware implementations, with experimental data showing that their benefits become more pronounced in smaller configurations. When examining test classification accuracy as a function of the number of network parameters, delays also enable more compact models while simultaneously improving accuracy. Axonal delays provide the largest increase in test classification accuracy per additional model parameter; however, the finer temporal granularity afforded by synaptic delays consistently yields higher accuracy, particularly in sparse configurations.

Three-factor learning rules also facilitate training of SRNNs. Considering the recurrent SRNNs trained on the SHD dataset (rows seven to nine of Table 1), weights-only models establish a baseline accuracy of 85.72% in fully connected configurations, which is 6.3% higher than that of SNNs. By adding input and recurrent delays test classification accuracies increase and become comparable to those of SNNs. Under sparse conditions, the accuracy loss is generally lower. To investigate the negligible impact of delays on SRNN test classification accuracy, the ablation experiments in Figure 5 isolate the contributions of input and recurrent delays. Introducing synaptic input delays or recurrent delays improves accuracy by 6.56% and 5.2%, respectively, whereas combining both yields a 6.6% improvement, indicating no additional benefit over feedforward delays. Axonal delays exhibit a similar trend, with an average accuracy that is 0.5% lower. In sparse configurations, adding recurrent delays provides a modest improvement of up to 1.3% in test classification accuracy compared to using input delays alone. These results highlight that increasing the number of model parameters without extending it's depth yields diminishing returns on the SHD dataset.

The SSC dataset presents a more challenging keyword-spotting task. Introducing learnable delays to feedforward SNNs (rows 10 and 11 of Table 1), substantially improves accuracy, yielding gains of 29.7% with BP and 19.67% with our three-factor learning model. When both weights and delays are co-learned, the classification accuracy of SNNs becomes comparable to offline implementations. In contrast, the effect of learnable delays on the SRNN is much smaller: adding synaptic and axonal delays increases accuracy by only 2.8% and 3%, respectively, even though the SRNN has three times more parameters. This suggests that we are reaching the feature-extraction limits of a single recurrent layer. The larger number of classes and the variability of recording conditions in the SSC dataset likely require deeper architectures.

## 6 Comparison and discussion

### 6.1 Comparison

**Figure 4:**
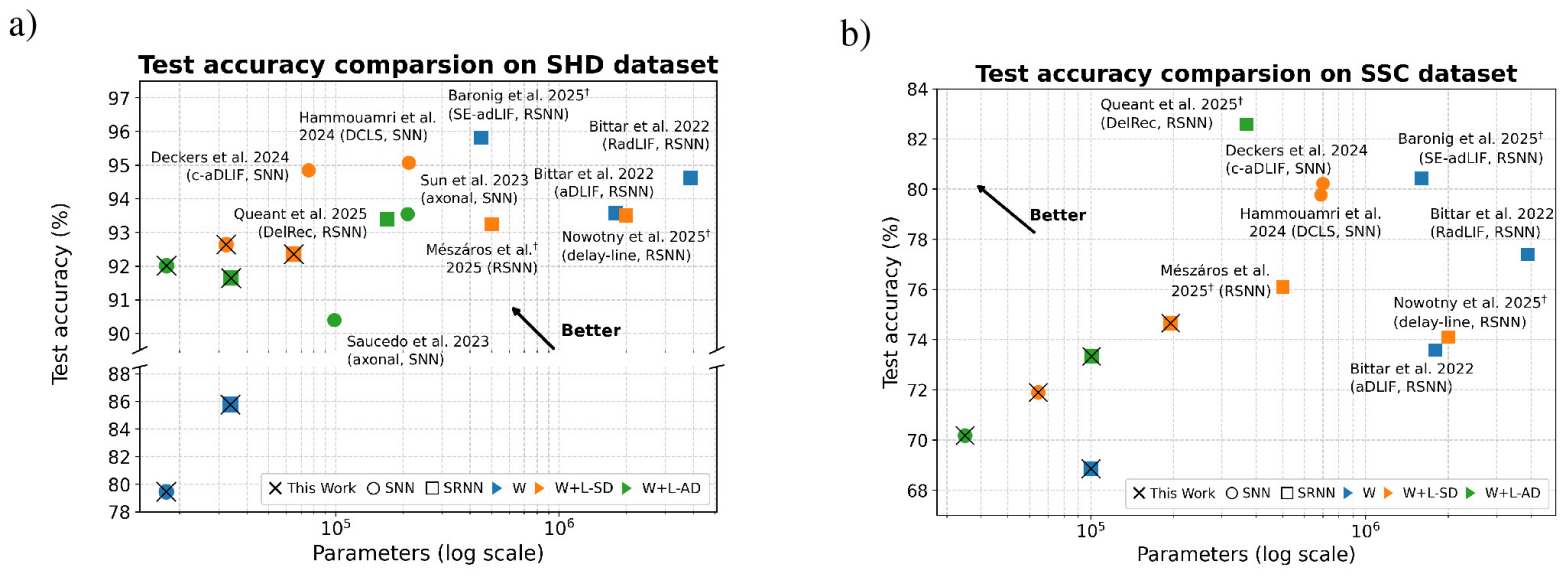
Comparison against offline methods on SHD and SSC. Marker shape distinguishes model type, whereas color determines the type and presence of delays. Markers overlaid with × correspond to our proposed online method. Datapoints annotated with ^†^ have employed a validation dataset to prevent potential overfitting to the test dataset. **(a)** Test accuracy comparision on SHD dataset. **(b)** Test accuracy comparision on SSC dataset

Figure 4 compares our online method (marked with × ) against state-of-the-art approaches on the SHD (4a) and SSC (4b) datasets, respectively.

On the SHD dataset, the highest reported accuracies are achieved by BPTT-based methods using two-layer SRNNs (Baronig et al., 2025) with adaptive LIF neurons and symplectic-Euler discretization, and two-layer feedforward SNNs (Hammouamri et al., 2024). Mészáros et al. (2025) also achieves near state-of-the-art accuracy with a single-layer SRNN trained with event propagation. Compared to Baronig et al. (2025)'s two-layer SRNN, our best-performing model achieves 3.4% lower task accuracy while reducing model size by 13.9 × . Compared to their small model of comparable size, the accuracy gap narrows to 2.2%. Relative to Hammouamri et al. (2024) our model incurs a 2.5% accuracy penalty with a 6.6 × smaller model while using a single delay layer rather than two, yielding also a 50% reduction in inference latency. It is noteworthy that adaptive thresholds or synaptic delays alone can approach state-of-the-art accuracy, which raises the question of whether further reductions in model size (Sun et al., 2025) or improvements in accuracy remain achievable. We also note that, unlike some competing approaches (Mészáros et al., 2025), we did not employ a validation set, which may have limited the robustness of our generalization estimates. Other competing methods achieve comparable accuracies on SHD using different architectures, such as adaptive SRNNs (Deckers et al., 2024; Bittar and Garner, 2022) or event-based state space models (Schöne et al., 2024; Soydan et al., 2024).

Although the SSC dataset is more challenging, we find the more versatile SRNN offers 3% higher accuracy, and therefore an advantage over feed-forward networks. Both DelRec (Queant et al., 2025) using BPTT-based offline training and event-propagation-based offline methods (Mészáros et al., 2025) report small but measurable gains for SRNNs with recurrent delays, echoing our finding. However, due to the larger number of classes and varying recording conditions, our method lags behind the SoTA by 7.9% (Queant et al., 2025) in classification accuracy. This result highlights that, as task complexity increases, deeper networks are required to enhance the representational capacity of the model. Consequently, the inability of our proposed method to scale to deeper networks becomes the primary limiting factor, and thus utilizing it with compact models may be more appropriate.

### 6.2 Parameter capacity experiments

**Figure 5:**
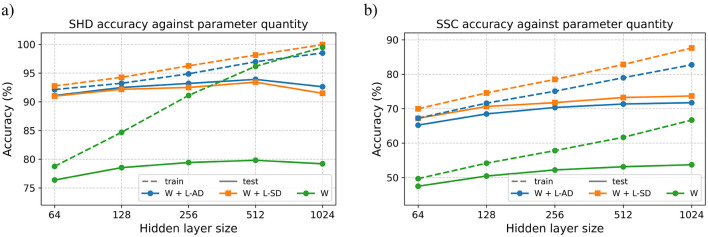
Test and training accuracy as a function of hidden layer width (64–1,024 neurons) for three model variants—weights-only (W), weights with synaptic delays (W + L-SD), and weights with axonal delays (W + L-AD)—evaluated on **(a)** SHD and **(b)** SSC. Dashed and solid lines denote training and test accuracy respectively.

To assess the performance limits of the proposed method, we vary the hidden layer size between 64 and 1,024 neurons for a single-hidden-layer feedforward SNN, reporting test accuracy on SHD and SSC. SRNNs are excluded from this analysis due to prohibitive memory requirements beyond 256 neurons. As shown in Figures 5a, b, increasing hidden layer width improves both train and test accuracies up to 256 neurons, beyond which test accuracy plateaus while training accuracy continues to rise, indicating overfitting. This pattern holds across both datasets and all model variants, with the exception of the weights-only (W) model on SHD, where saturation occurs earlier at 128 neurons. The growing train-test divergence beyond 256 neurons confirms that width is not an effective scaling axis in this regime, and that generalization rather than representational capacity is the primary constraint.

On SHD, the capacity analysis reveals that (W + L-AD) configuration with 256 neurons achieves 93.2% test accuracy, surpassing the (W + L-SD) configuration with 128 hidden neurons reported in the previous section. This brings our best result closer to the one-layer SoTA of 94.59% (Baronig et al., 2025), narrowing the gap to 1.4%. Two-layer architectures from Baronig et al. (2025) and Hammouamri et al. (2024) still report higher accuracies, with gaps of 2.6% and 1.9% respectively, while Mészáros et al. (2024) report comparable results albeit under ten-fold cross validation, which tends to yield more conservative estimates. On SSC, the recurrent model with synaptic delays outperforms all feedforward variants regardless of width, suggesting that recurrent connectivity contributes more to classification accuracy than model size. For eligibility propagation, which is practically limited to one hidden layer, this underscores that architectural versatility is a more effective path to improved generalization than increasing capacity.

This behavior is expected. Our method approximates the true gradient, and while we demonstrate parity with BPTT under identical configurations (Table 1), exact gradient methods (Hammouamri et al., 2024; Mészáros et al., 2025; Baronig et al., 2025) are likely to surpass approximate approaches on increasingly complex tasks. The advantage of the proposed method lies not in peak accuracy but in its ability to compute gradients in real time, substantially reducing the memory buffering requirements of BPTT and, to a lesser extent, EventProp, making it better suited for resource-constrained and online learning settings.

### 6.3 Efficacy of delay learning

**Figure 6:**
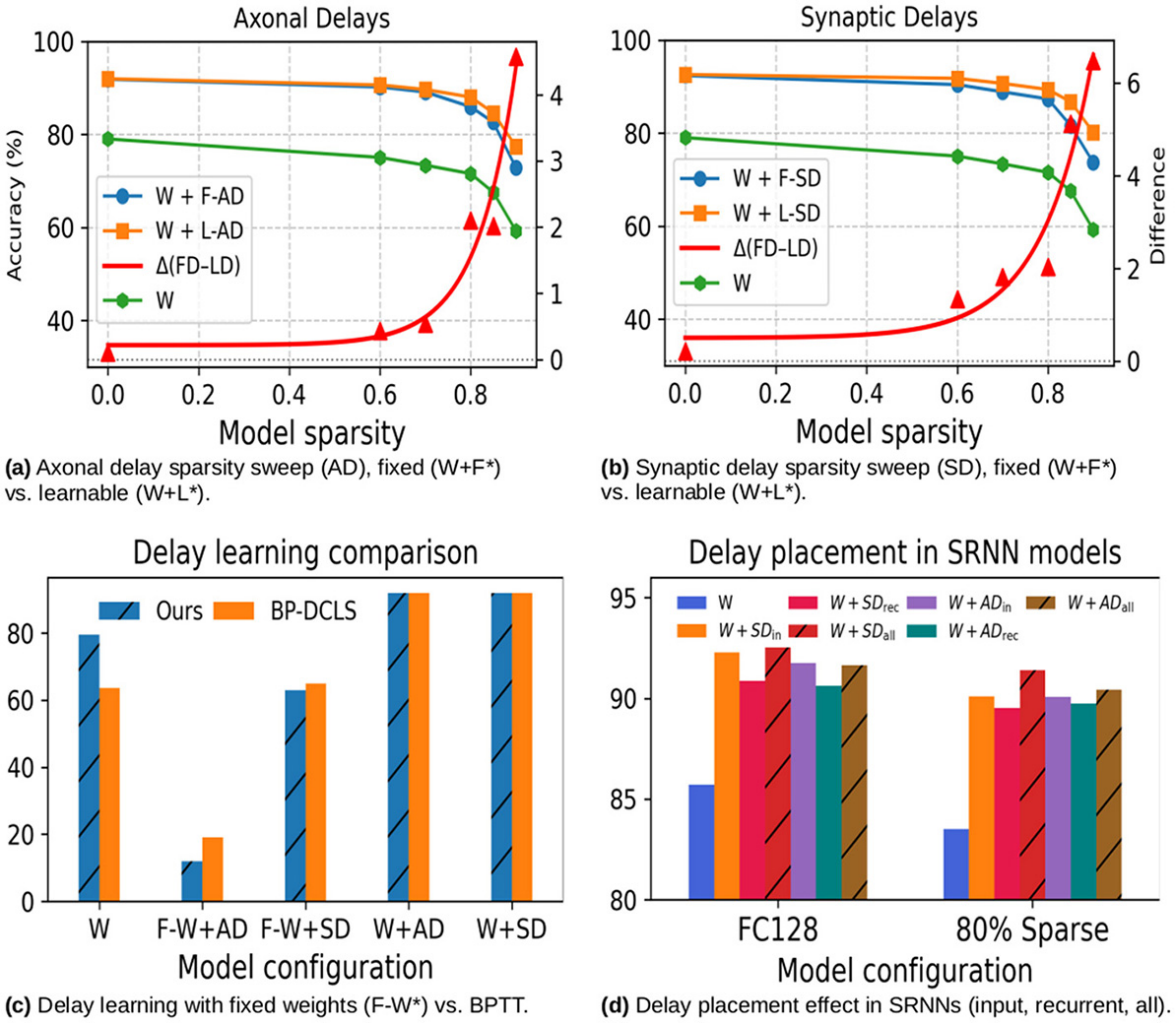
Control experiments illustrating the efficacy of delay learning. **(a)** Axonal delay sparsity sweep (AD), fixed (W+F*) vs. learnable (W+L*). **(b)** Synaptic delay sparsity sweep (SD), fixed (W+F*) vs. learnable (W+L*). **(c)** Delay learning with fixed weights (F-W*) vs. BPTT. **(d)** Delay placement effect in SRNNs (input, recurrent, all).

The major contribution to test classification accuracy is offered by the addition of fixed delays. As shown in Table 1, co-learning weights and delays in dense situations offers only a modest 1–2% accuracy improvement. Consequently, we conducted further experiments to better understand the impact of delay learning. The efficacy of delay learning becomes more apparent under highly sparse conditions, where contributions up to 6% over fixed delays can be observed, as shown in Figures 6a, b.

In control experiments comparing our method against BPTT, we observe that the three-factor delay learning rules are more effective for synaptic delays than for axonal delays. Comparing them to backpropagation in Figure 6c shows that the approximation holds for synaptic parameters with a gap of 2%, while for axonal parameters it starts to break down as the gap widens to 8%. The widened gap for axonal delays might be explained by the approximate parameter updates accumulating into large errors, since each axonal parameter at the input neuron is derived from the accumulated contributions of all post-synaptic neurons. The significant drop in classification accuracy for F-W+AD in Figure 6c is likely due to the small number of trainable parameters (116 in total). However, as learning under sparse conditions shows, when co-learned the impact of these errors could potentially have a regularization effect and overall yields acceptable results. These inaccuracies could potentially be mitigated by adopting alternative coding strategies, such as time-to-first-spike (TTFS) coding, which would allow axonal delays to be assigned locally to the hidden neuron rather than the input neuron.

### 6.4 On the on-chip implementation

Speech recognition systems benefit from on-device learning, allowing *in-situ* fine-tuning of network parameters to user-specific characteristics. However, edge-deployed neural networks are tightly constrained by power and memory, making any additional buffering—such as that required for delay elements—costly Frenkel and Indiveri (2022); Davies et al. (2018). On-device learning further increases memory demands. Here we show that for a fixed memory budget, structural sparsity can free enough capacity to accommodate both delay parameters, their associated buffers for implementation, as well as the temporary storage needed for parameter updates.

For sparsity levels in the range of 40–80%, this approach is suitable and achieves significant accuracy gains, on top of which, adaptability enabled by on-chip learning can provide an additional 3% improvement as discussed in Section 6.3. Axonal delays scale linearly while synaptic delays scale quadratically with the layer size, which impacts the memory requirements for both parameter storage and their implementation. Beyond the buffering requirements for delayed synaptic integration, the real-time calculation of delay parameter updates requires implementing the product between the eligibility trace and the surrogate kernel at the time the spike reaches the post-synaptic neuron.

When considering axonal delays, delaying the input spike train serves the dual purpose of implementing the delay and keeping track of the timestep at which the pre-synaptic spike influences the post-synaptic potential. Equation 14 gives the memory required


$$\begin{array}{c} M_{\text{axonal}}=n_{\text{in}}\cdot 2^{B_{d}}, \end{array}$$ (14)

where *n*_in_ is the input layer size and *B*_*d*_ is the delay resolution in bits. When considering synaptic delays, ring buffers are typically employed to implement the delay; however, when this mechanism is used, the timing of the pre-synaptic spike is lost, and must therefore also be tracked separately. The memory requirement amounts to the cost of delaying the input spike train and the ring buffer that accumulates the delayed synaptic current. This can be calculated with Equation (15),


$$\begin{array}{c} M_{\text{synaptic}}=n_{\text{in}}\cdot 2^{B_{d}}+n_{\text{hidden}}\cdot 2^{B_{d}}\cdot B_{v}, \end{array}$$ (15)

where *n*_hidden_ is the size of the hidden layer and *B*_*v*_ is the membrane potential resolution in bits. Finally, the buffering of the eligibility trace is required in both scenarios, with a memory cost proportional to the size of the surrogate gradient kernel, as defined in Equation 16


$$\begin{array}{c} M_{\text{trace}}=n_{\text{hidden}}\cdot k\cdot B_{v}, \end{array}$$ (16)

where *k* is the size of the look-up table holding the surrogate gradient.

Considering a typical quantization of the setup in Section 4.2 (a network with a 116 × 128 hidden layer and a 128 × 20 readout layer), with 8-bit weights, 5-bit delays, and 16-bit membrane potentials, we now account for the memory cost of implementing delays and their learning. For reference, the weights-only baseline has a total memory footprint of 141.632k bits. A model with learnable axonal delays requires *M*_axonal_+*M*_trace_ = 28.288k bits to support delay learning, leaving 113.344k bits for parameters. Since the *n*_in_ axonal delay parameters are 5-bit values totalling only 580 bits, the vast majority of this budget is available for synaptic weights, and the model must be at least 20% sparse to consume the same memory footprint as a weights-only model. A model with learnable synaptic delays requires *M*_synaptic_+*M*_trace_ = 93.824k bits, leaving only 47.808k bits for parameters. Given that hidden-layer parameters are 13 bits and readout parameters are 8 bits, this model must be at least 78% sparse.

At these levels of sparsity, Figures 6a, b show that co-learned synaptic and axonal delays offer 7–13% higher classification accuracy on the SHD dataset. Thus, for a fixed memory footprint, implementing learnable delays on chip not only provides higher classification accuracy compared to a weights-only baseline, but also enables *in-situ* parameter updates. As research provides evidence of the utility of delays and heterogeneous parameter sets for yielding compact models (Sun et al., 2025), co-designing online-learning with quantization, and pruning offers a promising direction for future research.

## 7 Conclusion

In this work, we proposed three-factor learning rules for synaptic and axonal delays in SNNs and SRNNs and validated their implementation against state-of-the-art BPTT-based learning methods. We demonstrated their competitive performance, and studied the efficacy of delay learning through ablation experiments. We showed that the presence of delays improves the classification accuracy by up to 18% in SNNs and 7.9% in SRNNs. We also showed that treating the delays as learnable parameters is only beneficial in highly sparse networks where they can contribute up to 6% to classification accuracy. Although small, the gain in classification accuracy can benefit low power edge-oriented applications substantially, specially those requiring adaptation to user specific features. Further research is required to address the challenge of scaling local learning rules to deeper architectures. By enabling on-device learning and maintaining competitive accuracy, three-factor delay learning rules show good promise in becoming a pragmatic solution for SWaP-constrained embedded systems.

## Data Availability

Publicly available datasets were analyzed in this study. This data can be found here: The SHD and SSC datasets used in this study are publicly available online and were used with respect to their original license. They can be found at https://zenkelab.org/resources/spiking-heidelberg-datasets-shd.
