# Supplementary material for: Three factor delay learning rules for spiking neural networks
Source: Front Neurosci. 2026 May 20;20:1814505. doi: 10.3389/fnins.2026.1814505 (PMC13229852; doi:10.3389/fnins.2026.1814505)
Supplement: Supplementary file 1 [file Data_Sheet_1.pdf]

Three factor delay learning rules for spiking neural networks - Supplementary Information

Luke Vassallo, and Nima Taherinejad

March 2026

1 Hyperparameters

The complete set of hyperparameters used for online and offline learning is provided in Table 1. Our three-factor delay learning rules do not utilize learning-rate schedulers. For delay learning, the Gaussian kernel used to compute delay parameter updates has a fixed $\sigma = 1.0$. In contrast, the Gaussian kernel employed in dilated convolution with learnable spacings (DCLS)-based offline learning reduces its variance proportionally with the epoch. Specifically, at the beginning of training, a large variance is applied, which gradually decreases throughout training. Ultimately, the Gaussian kernel converges to a binary profile resembling a spike.

Table 1: Hyperparameters for online learning with three-factor learning rules and offline DCLS-based configurations

**Parameter**	**Online (three-factor)**	**Offline (DCLS)**
trials	5	5
seed values	7270, 15795, 860, 5390, 13418	same
epochs (SHD, SSC)	60, 60	60, 60
learning rate (weights, delays)	$10^{-4}, 10^{-2}$	$10^{-3}, 10^{-2}$
initialisation (weights, delays)	kaiming normal, uniform	kaiming normal, uniform
optimiser (weights, delays)	adam, adam	adam, adam
learning rate scheduler	N/A	one cycle, cosine annealing
maximum delay $D_{\max}$	25 steps (250 ms)	25 steps (250 ms)
Gaussian parameters	$x = D_{ji},\ \sigma = 1.0$	$x = D_{ji},\ \sigma = 0.23^{1/\text{epoch}}$
time step Δt	10 ms	10 ms
membrane time constant τ_m	20 ms	20 ms
membrane time constant τ_o	1000 s	1000 s
